# Linking Defect-Controlled Grain Growth and Band-Edge Optical Response in Chymosin-Assisted Pechini-Derived CeO_2−δ_ Nanoparticles

**DOI:** 10.3390/ma18235282

**Published:** 2025-11-23

**Authors:** Maria Suêd M. Assis, Jorge A. V. Gonçalves, Robert S. Matos, Nilson S. Ferreira

**Affiliations:** 1 Department of Physics, Federal University of Sergipe, São Cristóvão 49100-000, SE, Brazil; mariasued23@academico.ufs.br; 2 Department of Agricultural Engineering, Federal University of Sergipe, São Cristóvão 49100-000, SE, Brazil; javgdbg@gmail.com; 3 Amazonian Materials Group, Federal University of Amapá, Macapá 68911-477, AP, Brazil; robert_fisic@unifap.br

**Keywords:** CeO_2−δ_ nanoparticles, chymosin, Pechini synthesis, oxygen vacancies, grain growth kinetics, Urbach energy

## Abstract

We investigate how grain growth, strain relaxation, and vacancy chemistry shape the near-edge optical response of nanocrystalline Ce$O_{2-\delta }$ prepared by a chymosin-assisted Pechini route from nitrate–citrate precursors. Rietveld line-profile analysis shows that phase-pure Ce$O_{2-\delta }$ forms after calcination between 400 and 1000 °C. Over this range, the average crystallite size increases from ≈3.4 to ≈57 nm, while the microstrain decreases from 0.79% to 0.05%, with size–strain scaling consistent with interface-controlled grain growth that follows a normal growth law with exponent m=2 and activation energy Q≈155 kJ ${\mathrm{mol}}^{-1}$. Raman spectroscopy tracks the sharpening of the $F_{2g}$ mode and the fading of defect-related bands, X-ray photoelectron spectroscopy reveals a nonmonotonic evolution of the surface ${\mathrm{Ce}}^{3+}$ fraction and separates lattice from adsorbed oxygen species, and electron paramagnetic resonance detects vacancy-bound ${\mathrm{Ce}}^{3+}$ polarons that weaken at high temperature. Diffuse-reflectance UV–Vis spectra show a modest blue shift of the apparent band gap from $E_{g}\approx 2.78$ to 2.95 eV as crystallites coarsen, while the Urbach energy $E_{u}$ follows the ${\mathrm{Ce}}^{3+}$ content and sub-gap tailing. The structural, spectroscopic, and optical results together map out a quantitative connection between grain growth, vacancy populations, and near-edge optical properties in ${\mathrm{CeO}}_{2-\delta }$ nanoparticles.

## 1. Introduction

Nanocrystalline cerium oxide, ${\mathrm{CeO}}_{2}$, plays a central role in modern materials science, enabling advances in catalysis, electrochemical energy storage and conversion, environmental remediation, optoelectronics, and emerging biomedical technologies [1,2,3,4,5]. Across these domains, macroscopic performance is governed by a coupled landscape of point defects, lattice response, and morphology. Oxygen vacancies and the associated ${\mathrm{Ce}}^{3+}$/${\mathrm{Ce}}^{4+}$ redox couple set oxygen-storage capacity, surface reactivity, and oxygen-ion transport. Their mobility and clustering are tuned by dopant chemistry, nonstoichiometry, and strain [6,7,8,9,10]. The dielectric response correlates with these defects through grain size, film density, and processing environment, which together govern permittivity, relaxation, and leakage in high-*k* ceria and heterostructures [11]. At the nanoscale, morphology gates activity and transport because size and exposed facets modulate vacancy stability and reaction pathways, producing pronounced shape effects in ${\mathrm{CeO}}_{2}$ and supported catalysts [12,13,14,15]. This triad provides the framework for interpreting the structure–property trends pursued here.

Recent work continues to elevate ${\mathrm{CeO}}_{2}$-supported systems in carbon-management catalysis, including Ni/${\mathrm{CeO}}_{2}$ for ${\mathrm{CO}}_{2}$ methanation, and expands biomolecule-coordinated syntheses and oxide–nanocomposite sensing platforms that motivate scalable and defect-tunable routes [16,17]. Interpreting such advances requires a defect-resolved view because particulate ensembles can introduce wavelength-dependent multiple scattering, clustering, and local-field effects that obscure intrinsic electronic absorption. In practice, performance reflects coupled variations in particle size and morphology, oxygen-vacancy content (δ) and spatial distribution, and surface chemical state, including the ${\mathrm{Ce}}^{3+}$/${\mathrm{Ce}}^{4+}$ ratio and hydroxylation [18,19,20]. At the photochemical level, several mechanism families can couple to these descriptors in oxide nanocrystals: (i) defect-mediated photo-redox pathways in which ${\mathrm{Ce}}^{3+}$–$V_{O}$ states tailor sub-gap absorption and recombination; (ii) dielectric (Mie) resonances in high-index particulates that reshape local fields and apparent cross sections; (iii) phonon- and surface-mediated nonradiative channels that broaden the near-edge tail (Urbach energy) [21,22,23,24].

Traditional routes to ${\mathrm{CeO}}_{2}$ nanoparticles include hydrothermal and solvothermal syntheses, combustion methods, microemulsions, and Pechini-type polymeric precursors [25]. These approaches have enabled major progress, yet some sol–gel and combustion variants rely on metal alkoxides or narrow processing windows that force trade-offs between size control, surface chemistry, and production scale. Bioinspired sol–gel strategies have emerged as lower-cost and lower-impact alternatives that use multidentate organic ligands and natural chelators to coordinate metal cations and guide condensation [26]. Proteic sol–gel variants extend this concept by employing nutrient-rich media to complex metal salts and form oxides without specialized equipment [27,28], although feedstock perishability and compositional variability complicate storage, logistics, and standardization [27,29]. What is missing is a salt-based synthesis that pairs the economy and compositional simplicity of classical polymeric precursors with the chemical definition of a biomolecule in order to regulate nucleation, size, and vacancy formation at modest temperatures with reproducible logistics. Prior evidence that carbohydrate scaffolds can act as chelating and size-limiting agents in nitrate–citrate sols supports this direction [30]. More generally, proteins provide peptide backbones and side chains with carboxylate and carbonyl donors that chelate multivalent cations, steer condensation, and influence the combustion and mass-loss profile during calcination [31,32]; whey-protein-templated ZnO syntheses illustrate such control over nucleation and morphology [33].

We therefore address this gap with a chymosin-assisted Pechini route that preserves the economy of nitrate–citrate precursors while introducing a compositionally defined biomolecular coordination environment. Chymosin is an aspartic protease available in a lyophilized, compositionally defined form. In the mildly acidic citrate medium typical of Pechini syntheses, its peptide backbone and side chains offer abundant carboxylate and carbonyl donors, while histidine and lysine residues provide additional coordination sites. Lanthanide–peptide complexes bind through carboxylate and amide carbonyl groups, and coordinated states are distinguished from physical mixing by diagnostic shifts in asymmetric and symmetric ${\mathrm{COO}}^{-}$ stretches and in amide I/II bands [34]. For cerium, ${\mathrm{Ce}}^{3+}$ forms comparatively strong inner-sphere complexes with multidentate carboxylates in water [35]. Upon heating, the protein denatures and decomposes in a narrow temperature window, yielding a reproducible mass-loss and combustion profile that promotes uniform resin removal and controlled nucleation. These conditions favor a regulated oxygen-vacancy content in ${\mathrm{CeO}}_{2-\delta }$, where the evolution of ${\mathrm{Ce}}^{4+}$ and the emergence of ${\mathrm{Ce}}^{3+}$ features provide fingerprints of mixed valence in ceria nanostructures [36]. In this context, chymosin acts as a multidentate organic complexant through the same carboxylate and carbonyl donor motifs widely reported for Pechini-type and peptide–lanthanide systems [34,37,38].

Here we report the synthesis and characterization of nanocrystalline ${\mathrm{CeO}}_{2-\delta }$ prepared by a chymosin-assisted Pechini route that uses off-the-shelf nitrate salts and citrate–ethylene glycol chemistry. The process yields phase-pure ${\mathrm{CeO}}_{2-\delta }$ nanoparticles after calcination in the 400–1000 °C range, and it provides a chemically defined path to couple cation complexation and burn-out with crystallite formation and vacancy engineering.

## 2. Materials and Methods

### 2.1. Synthesis

${\mathrm{CeO}}_{2-\delta }$ nanoparticles were synthesized via a polymeric-precursor route assisted by an auxiliary protein chelator. Analytical-grade reagents were used as received without further purification: Ce(${{\mathrm{NO}}_{3})}_{3}\cdot$ ${\mathrm{6H}}_{2}$O (99.999%, Sigma-Aldrich), citric acid (≥99.5%, Sigma-Aldrich), ethylene glycol anhydrous (99.8%, Sigma-Aldrich), and chymosin (protein content ≥ 40%, specific activity ≥ 20 units ${\mathrm{mg}}^{-1}$ protein, Sigma-Aldrich). In a typical procedure, Ce(${{\mathrm{NO}}_{3})}_{3}\cdot$ ${\mathrm{6H}}_{2}$O was dissolved in deionized water and complexed with citric acid (0.1 g ${\mathrm{mL}}^{-1}$) at a CA:Ce molar ratio of 3:1. The pH of the solution was adjusted to 6.5 using aqueous ammonia. Subsequently, a chymosin solution was added at 40 °C to provide approximately 20 mg of protein per mmol of Ce. The mixture was magnetically stirred at approximately 600 rpm for 30 minutes at 40 °C to ensure homogeneous complexation of Ce ions with peptide and citrate ligands before resin formation. Ethylene glycol was then added to achieve an ethylene glycol to citric acid (EG:CA) mass ratio of 4:1. The resulting sol was concentrated at 90 °C under continuous stirring until a viscous polymeric resin formed. This resin was dried for 12 h at 120 °C to obtain a xerogel, which was subsequently calcined at 400–1000 °C for 2 h to yield ${\mathrm{CeO}}_{2-\delta }$ nanoparticles.

### 2.2. Characterization

The formation and crystalline phase purity of the ${\mathrm{CeO}}_{2-\delta }$ nanoparticles were confirmed by powder X-ray diffraction (XRD) using a Bruker D8 Advance diffractometer with Cu Kα radiation (λ=0.15406 nm), operated at 40 kV and 40 mA over the 2θ range of $20^{{}^{\circ}}$–$80^{{}^{\circ}}$ with a step size of $0.02^{{}^{\circ}}$. Instrumental broadening was determined using a silicon standard (Si SRM 640d, NIST), and peak profiles were fitted using WinPLOTR [39]. The angular dependence of the full width at half maximum (FWHM) was described by the Caglioti relation [40]. Powder diffraction patterns were refined with FullProf [41], and peak profiles were described using the Thompson–Cox–Hastings (TCH) modified pseudo-Voigt function [42]. In practice, we refined a global scale factor, a zero shift, a fourth-order polynomial background, lattice parameters, profile-shape and width terms (five in total), a global isotropic displacement parameter, two axial-asymmetry terms, site occupancies, and the pseudo-Voigt mixing fraction. Parameters were updated iteratively by least squares [41] until the difference between observed and calculated intensities was minimized. Microstructural parameters were obtained from the analysis of anisotropic peak broadening. We examined the broadening of the (0k0) reflections relative to other directions to gauge anisotropy in crystallite size and microstrain. In the peak profile, the Lorentzian term was associated with finite-size effects and the Gaussian term with strain. Thus, size anisotropy was described with a spherical-harmonics model [43]:

(1) $$\beta_{hkl}=\frac{\lambda }{D_{hkl}\cos\theta }=\frac{\lambda }{\cos\theta }\sum_{lmp}a_{lmp}\;y_{lmp}\;\left(\Theta_{hkl},\Phi_{hkl}\right),$$

where $\beta_{hkl}$ is the integral breadth of the (hkl) reflection, $y_{lmp}\left(\Theta_{hkl},\Phi_{hkl}\right)$ are real spherical harmonics (with $\Theta_{hkl}$ and $\Phi_{hkl}$ the polar and azimuthal angles of the [hkl] vector in a crystallographic Cartesian frame), and $a_{lmp}$ are refinable coefficients set by the Laue class ($Fm\bar{3}m$ here) [44]. After refining the $a_{lmp}$ coefficients, we calculated the direction-dependent crystallite size $D_{hkl}$ and the microstrain using instrumental parameters *U*, *V*, and $W_{\mathrm{instr}}$ from an externally measured resolution file. Strain anisotropy was modeled from the variance of the quartic form $M_{hkl}$ in reciprocal space [45]:

(2) $$\sigma^{2}\;\left(M_{hkl}\right)=\sum_{\begin{array}{c} H,K,L\geq 0\\ H+K+L=4 \end{array}}S_{HKL}\;h^{H}k^{K}l^{L}.$$

where the number of refined $S_{HKL}$ coefficients depends on the crystalline symmetry. Here, “anisotropy” refers to the departure from the average apparent size when probed along different directions in reciprocal space. The standard deviation around the global average apparent size therefore quantifies the degree of anisotropy and should not be interpreted as an uncertainty in the mean size.

The formation of defective ${\mathrm{CeO}}_{2}$ nanoparticles was further confirmed by Raman spectroscopy. Raman spectra were acquired on a Bruker SENTERRA II spectrometer with 532 nm excitation in the high-resolution configuration. Sample composition and surface chemistry were examined by X-ray photoelectron spectroscopy (XPS) using a Thermo-VG Scientific ESCALAB 250 system with Al Kα radiation (1486.5 eV). Electron paramagnetic resonance (EPR) spectra were recorded at 300 K on an X-band Bruker ELEXSYS E580 spectrometer, with temperature control provided by an Oxford ITC503 unit. Spectra were collected using a modulation frequency of 100 kHz, a modulation amplitude of 0.2 mT, and a microwave power of 1 mW. Diffuse-reflectance UV–vis spectra of ${\mathrm{CeO}}_{2}$ powders were recorded at room temperature on a Shimadzu UV-3600 fitted with an integrating sphere, using ${\mathrm{BaSO}}_{4}$ as the reflectance standard. Raw signals were converted to absolute reflectance

(3) $$R\left(E\right)=\frac{S(E)-D(E)}{R_{\mathrm{std}}\left(E\right)-D\left(E\right)},$$

with *S* as the sample signal, $R_{\mathrm{std}}$ the ${\mathrm{BaSO}}_{4}$ reference, and *D* the dark background. After converting the spectra to the reflectance–absorbance ordinate $A_{R}\left(E\right)=-\log_{10}R\left(E\right)$ as an effective diffuse-field attenuation, we minimized multiple-scattering bias by using a consistent $A_{R}$ workflow and by cross-validating edge positions with both Tauc and derivative estimators. Photon energies were obtained from E[eV]=1240/λ[nm], and a small pre-edge offset was removed by subtracting the average $A_{R}$ over a fixed pre-edge window. All analysis was confined to 2.0–6.2 eV (200–620 nm). To stabilize derivatives and linear fits without distorting the edge, spectra were lightly smoothed with a Savitzky–Golay filter (50–100 meV window; polynomial order ≤3). The edge energy $E_{\mathrm{edge}}$ was taken as the most prominent positive extremum of $dA_{R}/dE$ within 2.0–6.2 eV. Apparent gaps were obtained from Tauc plots of ${\left[A_{R}\left(E\right)\;h\nu \right]}^{n}$ versus E=hν with n=2 (direct allowed) and $n=\frac{1}{2}$ (indirect allowed) [46,47]. Linear segments were chosen on the rising edge between 5–60% of the ordinate maximum while remaining within the stated energy range. We fitted Y=mE+b by ordinary least squares and took the intercept $E_{g}=-b/m$ as the apparent gap, reporting 95% confidence intervals from the regression covariance. To choose between direct and indirect models, we combined optical diagnostics with statistical criteria and simple predictive checks: corrected Akaike and Bayesian information criteria for the two-parameter linear fit [48,49], five-fold cross-validated root-mean-square error (RMSE) within the fitted window, and a bootstrap in which the fit window was jittered (≥200 resamples). Fits that disagreed with the derivative-based edge were penalized by $\left|E_{g}-E_{\mathrm{edge}}\right|$. The Urbach tail was obtained from a linear fit of $\ln A_{R}$ versus *E* over $\left[E_{\mathrm{edge}}-0.40,\;E_{\mathrm{edge}}-0.05\right]$ eV; the slope gives ${\left(E_{u}\right)}^{-1}$, with a high $R^{2}$ indicating a sensible exponential tail near the band edge [50].

Reported confidence intervals reflect within-batch statistical uncertainty obtained by bootstrap resampling of the Arrhenius fits, while using a common instrument-resolution file and a fixed TCH/Popa line-profile model across all refinements. These intervals capture fit and instrument contributions; they do not include between-batch variance. For scale-up, we plan a multi-batch study (at least three independent syntheses) and will report inter-batch relative standard deviations (%RSD) for *D*, $E_{g}$, $E_{u}$, and *Q*, computed as $\%RSD=100\times \frac{s}{\bar{x}}$, where *s* is the sample standard deviation across batches and $\bar{x}$ is the batch mean for the metric of interest.

## 3. Results and Discussion

**Figure 1:**
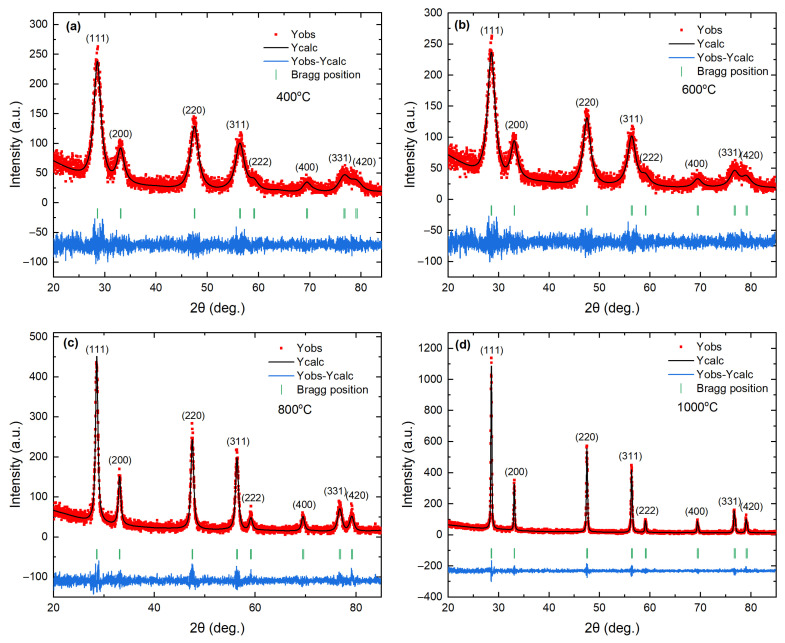
Observed (red), calculated (black), and differential patterns from Rietveld refinement patterns of X-ray diffraction for ${\mathrm{CeO}}_{2-\delta }$ nanoparticles calcined at (**a**) 400 °C, (**b**) 600 °C, (**c**) 800 °C, (**d**) 1000 °C. The red square symbols and the black line denote the observed and calculated intensities, respectively. Short vertical green lines indicate the positions of the possible Bragg reflections in the $Fm\bar{3}m$ structure. The difference between the observed and calculated profiles is shown in blue at the bottom.

Figure 1a–d shows Rietveld refinement profiles of the powder XRD patterns for samples calcined at 400, 600, 800, and 1000 °C. All diffraction peaks can be indexed to fluorite-type ${\mathrm{CeO}}_{2}$ (space group $Fm\bar{3}m$, ICSD #24887) with the expected (111), (200), (220), (311), (222), (400), (331), and (420) reflections and no additional peaks within the detection limit. The refinements employed the standard fluorite model, with cations on the 4a site (0,0,0) and oxygen on the 8c site $\left(\frac{1}{4},\frac{1}{4},\frac{1}{4}\right)$. The refined structural and microstructural parameters are summarized in Table 1. The reduced $\chi^{2}$ values range from 1.06 to 1.15, indicating statistically consistent single-phase fits for all samples. A small yet meaningful evolution of the lattice parameter accompanies thermal treatment. Relative to ${\mathrm{CeO}}_{2-\delta }$ nanoparticles calcined at 400 °C (*a* = 5.41489(4) Å), the lattice contracts to a minimum at 800 °C $\left(a=5.41188(2)\;Å\right)$ and then increases slightly at 1000 °C $\left(a=5.41337(2)\;Å\right)$. The net change from 400 to 1000 °C is Δa=−0.00152Å, which corresponds to about −0.028%. This lattice change of −0.00152 Å is ∼34σ larger than the combined one-sigma uncertainty of the two endpoints, confirming that the contraction is significant relative to the Rietveld statistical error. The unit cell volume follows the same nonmonotonic trend, decreasing from V=158.77(4) to 158.51(2) and then increasing to $158.64\left(2\right)\;{Å}^{3}$. The lattice volume change when the calcination temperature is increased from 400 to 1000 °C is $\Delta V=-0.13\;{Å}^{3}$, which corresponds to approximately −0.082%. These shifts exceed the standard uncertainties for each data point, so they are unlikely to arise from a refinement artifact. The overall contraction relative to the ${\mathrm{CeO}}_{2-\delta }$ nanoparticles calcined at 400 °C is consistent with defect recovery in ceria treated in air, where higher temperatures decrease the concentration of oxygen vacancies and the fraction of ${\mathrm{Ce}}^{3+}$, driving the lattice parameter toward the ${\mathrm{Ce}}^{4+}$ limit [6,51].

The average crystallite size of the ${\mathrm{CeO}}_{2-\delta }$ nanoparticles increases slightly from 〈D〉=3.43(4) to 3.98(2)nm as the calcination temperature increases from 400 to 600 °C. However, the growth becomes much more pronounced, from 14.9(3)nm to 57.3(6)nm, as the calcination temperature increases from 800 to 1000 °C. In parallel, the mean microstrain decreases overall from 0.79(2)% to 0.05(1)% in the calcination range of 400–1000 °C, with a local increase at a calcination temperature of 800 °C $\left[0.39(8)\;\%\right]$ relative to 600 °C $\left[0.17(3)\;\%\right]$. This size–strain anti-correlation is a classic signature in line-profile analysis. In our case, it likely reflects two coupled processes: grain coarsening becomes efficient once point defects become mobile, which increases 〈D〉; recovery and redistribution of oxygen vacancies and associated ${\mathrm{Ce}}^{3+}$ centers reduce local elastic fields, hence the overall drop in 〈ε〉 [6,51]. The small local maximum in strain for the ${\mathrm{CeO}}_{2-\delta }$ nanoparticles calcined at 800 °C is consistent with a transient regime in which rapid growth and defect rearrangement generate short-range heterogeneity before further relaxation in the ${\mathrm{CeO}}_{2-\delta }$ nanoparticles calcined at 1000 °C. Two technical points support the robustness of these trends. First, the absolute change in *a* is small, so the microstructural model used in the refinement must avoid over-fitting peak breadth. Here, the simultaneous decrease in 〈ε〉 and increase in 〈D〉 across many Bragg families argues against an instrument-driven artifact and favors a real microstructural evolution. Second, the progressive improvement of background-subtracted residuals with calcination temperature in Figure 1 is consistent with a reduction in diffuse scattering from defected near-surface regions as the crystallites coarsen.

**Figure 2:**
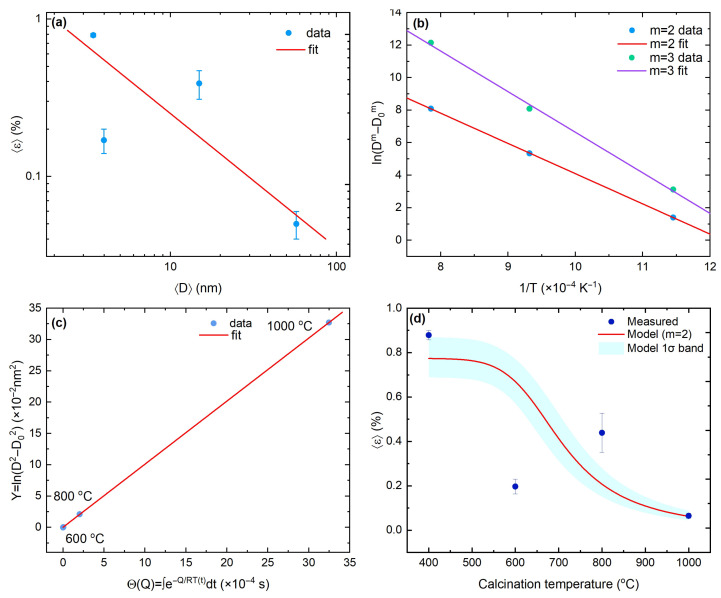
(**a**) Size–strain scaling $\langle \varepsilon \rangle =A\;{\langle D\rangle }^{-p}$. (**b**) Arrhenius transform $\ln\;\left(D^{2}-D_{0}^{2}\right)$ vs. 1/T; slope =Q/R; intercept $=\ln(K_{0}t)$; m=2 fit. (**c**) Master plot $D^{2}-D_{0}^{2}$ vs. $\Theta \left(Q\right)=\int \exp\;\left[-Q/(R\;T(t))\right]\;dt$ showing collapse across ramps/dwells. (**d**) Measured 〈ε〉 and coupled prediction $\langle \varepsilon \rangle \left(T\right)=A\;{\left[D_{0}^{2}+K_{0}\Theta \left(Q\right)\right]}^{-p/2}$; band is the 16–84th percentile from the joint covariances of (A,p) and $\left(D_{0},\ln K_{0}t,Q\right)$.

Thus, we further quantified the size–strain relation by fitting 〈ε〉 versus 〈D〉 to a power law on log–log axes (Figure 2a). The best-fit model is

(4) $$\langle \varepsilon \rangle \left(\%\right)\;=\;A\;{\langle D\rangle }^{-p},\;A=2.33\pm 0.20,\;p=0.895\pm 0.064,$$

which is consistent with interface-dominated or dislocation-mediated strain fields that scale with interfacial area ∝1/D and relax as grains coarsen. To move beyond a purely descriptive *D*–*T* view, we adopt the normal grain-growth law

(5) $$D^{m}-D_{0}^{m}\;=\;K_{0}\;t\;\exp\;\left(-\frac{Q}{RT}\right),$$

and evaluate the Arrhenius transform $\ln\;\left(D^{m}-D_{0}^{m}\right)$ versus 1/T [Figure 2b]. The data are essentially linear for m=2 and clearly inferior for m=3. For m=2, the fit yields $Q=155\pm 5\;\mathrm{kJ}\;{\mathrm{mol}}^{-1}$ with $R^{2}\approx 0.99$; enforcing m=3 increases the apparent barrier to $Q=210\pm 20\;\mathrm{kJ}\;{\mathrm{mol}}^{-1}$ and degrades the collapse. The preference for m=2, together with the lower *Q*, points to curvature-driven, interface-controlled grain-boundary migration as the rate-limiting step in nanocrystalline ${\mathrm{CeO}}_{2}$ [52], whereas higher apparent activation energies in coarser or porous bodies likely reflect drag and microstructural differences [53,54]. We further assessed the rate-limiting mechanism by performing Arrhenius fits of lnK versus 1/T for models with m=2 (interface-controlled) and m=3 (diffusion-limited). For m=2, the fit gives $Q=154.5\pm 0.6\;\mathrm{kJ}\;{\mathrm{mol}}^{-1}$ with a small residual (RMSE =0.019), whereas for m=3 it yields $Q=207\pm 10\;\mathrm{kJ}\;{\mathrm{mol}}^{-1}$ with poorer agreement (RMSE =0.310). Although mixed-control models cannot be resolved with only three temperatures, these results favor an interface-controlled grain-growth mechanism within the experimental uncertainty.

Non-isothermal schedules or unequal dwells are handled by the thermal budget

(6) $$\Theta \left(Q\right)=\int \exp\left[-\frac{Q}{RT(t)}\right]\;dt,$$

which collapses the transformed growth $Y\equiv D^{2}-D_{0}^{2}$ into a single straight line,

(7) Y=a+bΘ,

across the calcination range 600–1000 °C (Figure 2c), in line with a master-curve description under a single activated mechanism [55,56]. Because strain is empirically linked to size, the kinetics and the size–strain scaling combine into a predictive microstrain–temperature relation,

(8) $$\langle \varepsilon \rangle \left(T\right)=A{\left[D_{0}^{2}+K_{0}\Theta \left(Q\right)\right]}^{-p/2}.$$

Uncertainties are propagated by sampling (A,p) from the covariance of the log–log size–strain fit and $\left(D_{0},\ln K_{0}t,Q\right)$ from the covariance of the m=2 Arrhenius fit; we then report the pointwise median with a 1σ (16th–84th percentile) band. Its width at low *T* is governed mainly by $\ln K_{0}t$ (prefactor), while at high *T* it is dominated by *Q* (barrier). All high-temperature data (600–1000 °C) lie within the band; the 800 °C point sits near its upper edge and can be rationalized by mild anisotropic broadening or transient defect repartitioning during the onset of rapid growth, without requiring a change in mechanism (Figure 2d).

**Figure 3:**
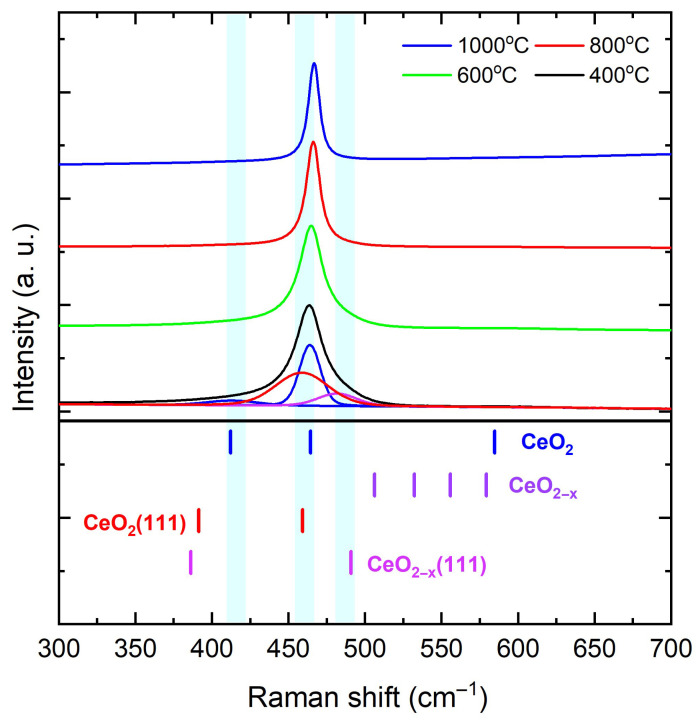
Room-temperature Raman spectra of ${\mathrm{CeO}}_{2-\delta }$ nanoparticles calcined at 400, 600, 800, and 1000 °C. Reference ticks mark bulk $F_{2g}$ and surface-related bands following Schilling et al. [59]. The $F_{2g}$ peak recenters at 465–466 ${\mathrm{cm}}^{-1}$ and narrows with calcination, while surface- and vacancy-associated features weaken.

Having established a quantitative size–strain relation, we turn to Raman spectroscopy to test whether the strain reduction tracks the healing of vacancy- and surface-related disorder. Figure 3 shows room-temperature Raman spectra of ${\mathrm{CeO}}_{2-\delta }$ nanoparticles calcined at 400, 600, 800, and 1000 °C. “Bulk-related” denotes the Γ-point Raman-active fluorite mode $F_{2g}$ of ${\mathrm{CeO}}_{2}$, observed at 465–466 ${\mathrm{cm}}^{-1}$ in large, well-oxidized crystallites, whereas “surface-related” refers to bands that arise from relaxation of momentum selection rules at nanocrystal surfaces or in defect-rich near-surface regions, as well as vacancy-assisted features [57,58,59,60,61]. The fluorite $F_{2g}$ mode dominates the spectrum, and with increasing calcination temperature its maximum shifts toward 465–466 ${\mathrm{cm}}^{-1}$ while its linewidth narrows, indicating reduced static disorder, a longer phonon correlation length, and partial relaxation of size- and nonstoichiometry-related strain [57,58,59]. For the ${\mathrm{CeO}}_{2-\delta }$ nanoparticles calcined at 400 and 600 °C, two additional low-energy components appear: a weak band near 433 ${\mathrm{cm}}^{-1}$ attributed to surface- and disorder-activated finite-*q* contributions to the $F_{2g}$ envelope [57,58], and a shoulder near 459 ${\mathrm{cm}}^{-1}$ consistent with a ${\mathrm{CeO}}_{2}$ (111) surface-related band [59,60]. A broader feature near 490 ${\mathrm{cm}}^{-1}$ is also observed and is assigned to a ${\mathrm{CeO}}_{2-\delta }$ (111) vacancy-assisted band [60]. Both the 459 and 490 ${\mathrm{cm}}^{-1}$ components diminish markedly with calcination, tracking the loss of defective or strongly expressed (111) terminations. Deconvolution of the asymmetric $F_{2g}$ envelope is treated as a lineshape model for a single Γ-point mode modified by confinement, surfaces, and defects rather than as multiple bulk eigenmodes: a finite-*q* low-frequency tail (∼430–440 ${\mathrm{cm}}^{-1}$), a bulk-like $F_{2g}$ center (465–466 ${\mathrm{cm}}^{-1}$), and a surface-related shoulder near ∼459 ${\mathrm{cm}}^{-1}$. The broader ∼490 ${\mathrm{cm}}^{-1}$ vacancy-assisted band is fitted separately [57,58,59,60]. The disorder-activated background in the 480–500 ${\mathrm{cm}}^{-1}$ range diminishes as the calcination temperature increases [57,59], and the vacancy-correlated band near 600 ${\mathrm{cm}}^{-1}$, typically enhanced in reduced ${\mathrm{CeO}}_{2}$ rich in ${\mathrm{Ce}}^{3+}$–$V_{O}$ motifs, is weak or not observed across the studied range [60,61]. In addition, the recentering and narrowing of $F_{2g}$, together with the attenuation of the 433, 459 [${\mathrm{CeO}}_{2}$ (111)], and 490 ${\mathrm{cm}}^{-1}$ [${\mathrm{CeO}}_{2-\delta }$ (111)] features, indicate that calcination promotes reoxidation, reduces the contribution from defect-rich (111) surfaces, and yields a more bulk-like fluorite Raman response [57,59]. These spectroscopic signatures are most consistent with intrinsic strain relief associated with oxygen-vacancy-related near-surface relaxation. Therefore, grain coalescence may facilitate the effect but is not its sole cause when considered together with the observed 〈ε〉 vs 〈D〉 scaling.

**Figure 4:**
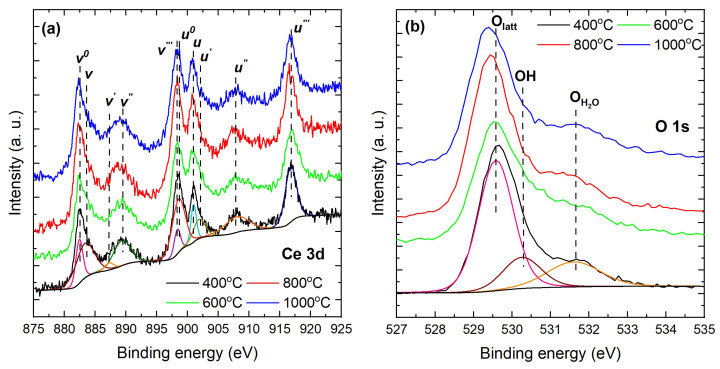
High-resolution XPS spectra of (**a**) Ce 3d and (**b**) O 1s for ${\mathrm{CeO}}_{2-\delta }$ nanoparticles calcined at 400–1000 °C.

Further insights into reduced near-surface disorder and vacancy healing, inferred via the surface ${\mathrm{Ce}}^{3+}$/${\mathrm{Ce}}^{4+}$ balance and vacancy-related signatures, were obtained from XPS measurements. All spectra for ${\mathrm{CeO}}_{2-\delta }$ nanoparticles calcined at different temperatures were aligned, sample by sample, to the 284.8 eV C 1s reference to avoid artificial trends from differential charging. A weak ∼289 eV carbonate component (O–C=O) was observed, consistent with air-exposed ${\mathrm{CeO}}_{2}$, and no N 1s signal was detected within instrumental limits. These routine surface species do not affect the Ce 3d/O 1s quantification of the ${\mathrm{Ce}}^{3+}$/${\mathrm{Ce}}^{4+}$ ratio used to assess vacancy chemistry. Figure 4a shows the Ce 3d spectra, which were deconvoluted into ten components associated with ${\mathrm{Ce}}^{3+}$ and ${\mathrm{Ce}}^{4+}$ final states [62,63]. The coexistence of both valence states across the series confirms the mixed-valence character of nanocrystalline ceria, consistent with established ceria redox chemistry [64,65]. For peak assignment, we followed the standard v/u notation, where *v* labels the ${\mathrm{3d}}^{5/2}$ features and *u* labels the 3$d^{3/2}$ features [62,63]. The pairs $\left(u_{0},v_{0}\right)$ and $\left(u^{\prime },v^{\prime }\right)$ arise from ${\mathrm{Ce}}^{3+}$ with predominant Ce(3$d^{9}$4$f^{2}$)O 2$p^{5}$ and Ce(3$d^{9}$4$f^{1}$)O 2$p^{6}$ character, respectively. The pairs (u,v), $\left(u^{\prime \prime },v^{\prime \prime }\right)$, and $\left(u^{\prime \prime \prime },v^{\prime \prime \prime }\right)$ correspond to ${\mathrm{Ce}}^{4+}$ with predominant Ce(3$d^{9}$4$f^{2}$)O 2$p^{4}$, Ce(3$d^{9}$4$f^{1}$)O 2$p^{5}$, and Ce(3$d^{9}$4$f^{0}$)O 2$p^{6}$ character, respectively. To ensure physically consistent fits, we constrained the spin-orbit splitting $\Delta_{\mathrm{so}}\left(u-v\right)$ within each doublet and enforced a common FWHM within a given chemical state [66,67].

The relative contributions of ${\mathrm{Ce}}^{3+}$ and ${\mathrm{Ce}}^{4+}$ were calculated as

(9) $$A_{{\mathrm{Ce}}^{3+}}=v_{0}+u_{0}+v^{\prime }+u^{\prime },\;A_{{\mathrm{Ce}}^{4+}}=v+v^{\prime \prime }+v^{\prime \prime \prime }+u+u^{\prime \prime }+u^{\prime \prime \prime },$$

with the fractional populations given by

(10) $${\mathrm{Ce}}^{3+}=\frac{A_{{\mathrm{Ce}}^{3+}}}{A_{{\mathrm{Ce}}^{3+}}+A_{{\mathrm{Ce}}^{4+}}},\;{\mathrm{Ce}}^{4+}=\frac{A_{{\mathrm{Ce}}^{4+}}}{A_{{\mathrm{Ce}}^{3+}}+A_{{\mathrm{Ce}}^{4+}}}.$$

The results are summarized in Table 2. For the ${\mathrm{CeO}}_{2-\delta }$ nanoparticles calcined at 400 °C, ${\mathrm{Ce}}^{3+}$ accounts for ∼35% of the total cerium signal (${\mathrm{Ce}}^{3+}$/${\mathrm{Ce}}^{4+}\approx 0.55$). The ${\mathrm{Ce}}^{3+}$ fraction decreases to ∼10% for nanoparticles calcined at 600 °C. This indicates that organics are fully removed and that oxygen transport is efficient, which reoxidizes ${\mathrm{Ce}}^{3+}$ to ${\mathrm{Ce}}^{4+}$ and heals oxygen vacancies; the result is a minimum surface-weighted ${\mathrm{Ce}}^{3+}$ fraction detected by XPS. For the ${\mathrm{CeO}}_{2-\delta }$ nanoparticles calcined at 800 °C, the ${\mathrm{Ce}}^{3+}$ fraction rises to ∼42%, and for those calcined at 1000 °C it approaches parity with ${\mathrm{Ce}}^{4+}$, (∼50%,$\;{\mathrm{Ce}}^{3+}/{\mathrm{Ce}}^{4+}\approx 0.98)$. This nonmonotonic evolution reflects the competition between defect annihilation at intermediate temperatures and oxygen-vacancy-driven reduction at higher temperatures, indicating that redistribution can enrich near-surface ${\mathrm{Ce}}^{3+}$ even as bulk-weighted probes indicate a cleaner, more oxidized lattice [64,65]. The O 1s spectra (Figure 4b) were deconvoluted into three physically justified components: $O_{\mathrm{Latt}}$ at 528.6–529.6 eV, assigned to lattice $O^{2-}$ in fluorite ${\mathrm{CeO}}_{2}$; $O_{\mathrm{OH}}$ at 531.3–531.8 eV, assigned to surface hydroxyls; and $O_{H_{2}O}$ at 532.7–533.2 eV, assigned to molecularly adsorbed water [64,68,69,70].

Oxygen stoichiometry was evaluated with two complementary approaches. From the ${\mathrm{Ce}}^{3+}$/${\mathrm{Ce}}^{4+}$ balance in Equation (11), the O/Ce ratio is

(11) $$x=\frac{\left[O\right]}{\left[Ce\right]}=\frac{3}{2}\;\left[{\mathrm{Ce}}^{3+}\right]+2\;\left[{\mathrm{Ce}}^{4+}\right],\;\left[{\mathrm{Ce}}^{3+}\right]+\left[{\mathrm{Ce}}^{4+}\right]=1,$$

whereas the O/Ce ratio obtained using only the lattice-oxygen area in O 1s is

(12) $$x^{\prime }=\frac{\left[O_{1s}^{\mathrm{Latt}}\right]}{\left[Ce_{3d}\right]}=\frac{A_{O,\mathrm{Latt}}}{A_{Ce}}\cdot \frac{S_{Ce}}{S_{O}},\;S_{O}=0.711,\;S_{Ce}=7.399,$$

with Scofield cross sections for Al Kα [71]. Restricting O 1s to the lattice component reduces the bias from hydroxyl and molecular water that are largely surface-derived [68,72,73]. Table 3 shows small thermally driven shifts in the O 1s components, with the lattice peak moving from 528.57 eV for the ${\mathrm{CeO}}_{2-\delta }$ nanoparticles calcined at 400 °C to approximately 529.3–529.4 eV for those calcined at 600–1000 °C, while $O_{\mathrm{OH}}$ and $O_{H_{2}O}$ remain near approximately 529.1–530.1 eV and 530.9–531.5 eV, respectively. These binding energy ranges are consistent with standard assignments in ${\mathrm{CeO}}_{2}$, where the lowest-BE O 1s component corresponds to lattice O near 529 eV, the intermediate component reflects hydroxyls near 531 eV, and the highest-BE feature reflects molecular water near 532 eV [68,72,74]. The lattice-oxygen fraction varies non-monotonically with calcination temperature: %$O_{\mathrm{Latt}}$ is 41.7% at 400 °C, decreases to 27.3% for ${\mathrm{CeO}}_{2-\delta }$ nanoparticles calcined at 600 °C, increases to 51.1% at 800 °C, and then stabilizes at 43.0% for those calcined at 1000 °C. In line with these variations, the Ce-balance method yields x=1.82, 1.87, 1.79, and 1.75 at 400, 600, 800, and 1000 °C, respectively, whereas the lattice-O method gives $x^{\prime }=2.27$, 2.09, 2.40, and 2.20 for the same sequence. The difference $\Delta =x-x^{\prime }$ is therefore negative at all temperatures, with Δ=−0.45 (400 °C), −0.22 (600 °C), −0.61 (800 °C), and −0.45 (1000 °C). The sign and trend of Δ indicate that the O 1s lattice-only route systematically returns larger O/Ce ratios than the Ce-balance route for these nanoparticles. Several non-exclusive factors can account for this: (i) component cross-talk in the 531–532 eV range, which can bleed surface OH/$H_{2}$O intensity into the lattice envelope during fitting and inflate $A_{O,\mathrm{Latt}}$ [72,73]; (ii) different effective sampling depths and matrix effects between O 1s and Ce 3d, since the higher kinetic energy Ce 3d electrons probe slightly deeper and the near-surface may appear more oxidized in O 1s than in Ce 3d [72,75]; (iii) uncertainties in Ce 3d multiplet deconvolution and inelastic background, which can bias the ${\mathrm{Ce}}^{3+}$ fraction used in Equation (11) [68,72]. The largest magnitude of |Δ| occurs for ${\mathrm{CeO}}_{2-\delta }$ nanoparticles calcined at 800 °C (|Δ|=0.61), coincident with the peak %$O_{\mathrm{Latt}}$ (51.1%) and the highest $x^{\prime }$ (2.40). This can be rationalized by defect reorganization near 800 °C that enhances oxygen uptake and strengthens the lattice-O signal relative to Ce 3d [76,77], together with temperature-dependent redox equilibria on ceria surfaces that modulate the balance of lattice O, hydroxyls, and adsorbed water [64]. For calcination at 600 °C, dehydroxylation and partial healing of vacancies commonly reduce surface species and can shift the balance toward higher *x*, while still leaving $x^{\prime }<x$ by a smaller margin [72,76]. At 1000 °C, further high-temperature reduction lowers *x* and decreases %$O_{\mathrm{Latt}}$, whereas $x^{\prime }$ remains higher; consequently, Δ<0, consistent with persistent near-surface oxygenated species and depth-weighting effects [64,68,76]. Therefore, both approaches capture the same qualitative thermal evolution of the oxygen chemistry, although the absolute O/Ce ratios invert the usual ordering ($x^{\prime }≲x$).

**Figure 5:**
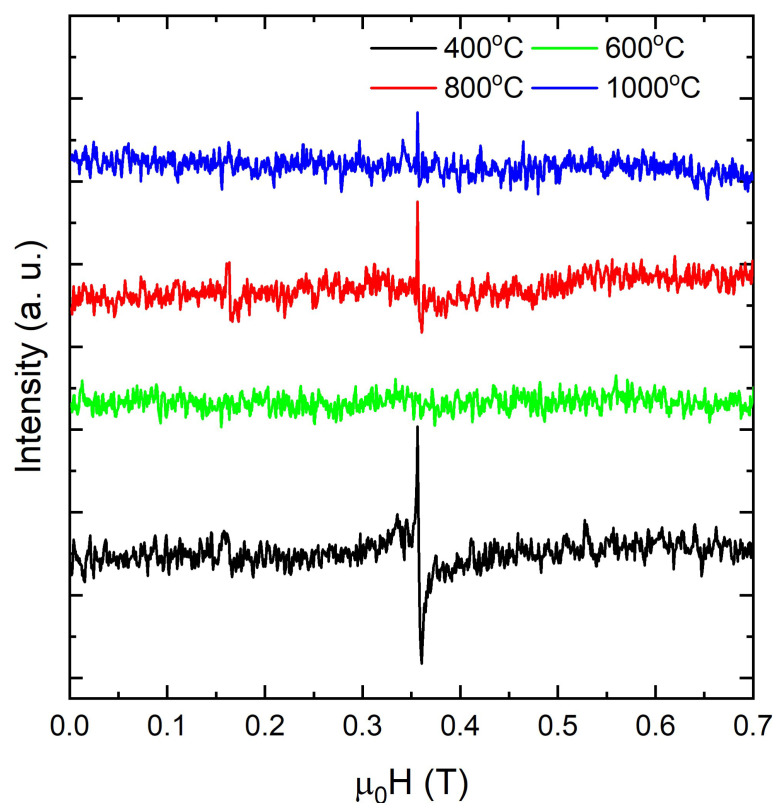
Room-temperature EPR spectra of ${\mathrm{CeO}}_{2-\delta }$ nanoparticles calcined at 400, 600, 800, and 1000 °C.

We now turn to EPR to probe how electronic and structural perturbations reshape the phonon spectrum of ${\mathrm{CeO}}_{2-\delta }$ nanoparticles. XPS provides an average chemical-state picture within its probing depth across calcination temperatures in the ${\mathrm{CeO}}_{2-\delta }$ nanoparticles but cannot determine whether lattice anomalies arise from ionic-size effects, from ${\mathrm{Ce}}^{4+}\to {\mathrm{Ce}}^{3+}$ reduction, or from the balance between local contraction near oxygen-rich coordinations and the chemical expansion driven by increasing $V_{O}$ with concomitant ${\mathrm{Ce}}^{4+}\to {\mathrm{Ce}}^{3+}$ reduction. At low calcination temperatures in air, the powders are more reduced, so partial ${\mathrm{Ce}}^{4+}\to {\mathrm{Ce}}^{3+}$ conversion and oxygen-vacancy formation chemically expand the fluorite lattice. At higher calcination temperatures, oxygen uptake oxidizes ${\mathrm{Ce}}^{3+}\to {\mathrm{Ce}}^{4+}$ and annihilates $V_{O}^{••}$, which limits further reduction and contracts the lattice toward the stoichiometric limit. To disentangle these possibilities, we use EPR, which is uniquely sensitive to the local redox environment. Figure 5 shows room-temperature EPR spectra (ν≈9.4–9.8GHz) collected for ${\mathrm{CeO}}_{2-\delta }$ nanoparticles calcined at 400–1000 °C. Each spectrum exhibits a single resonance at H≈0.34–0.36T, which corresponds to g≈1.95–1.97 with a centroid near g≃1.96. The line shape is a first-derivative feature well described by a Voigt-like profile. No hyperfine structure is resolved, as expected, because the abundant Ce isotopes have I=0. The spectra are essentially symmetric and show no Dysonian asymmetry, consistent with insulating powders.

At room temperature, ${\mathrm{Ce}}^{3+}$ centers in ${\mathrm{CeO}}_{2}$ exhibit an axial *g* tensor which, after powder averaging and motional effects, produces a single envelope centered at g≈1.95–1.97 [78,79]. The measured field positions, line shapes, and their evolution across the calcination series are consistent with ${\mathrm{Ce}}^{3+}$ stabilized in oxygen-deficient environments, including vacancy-bound ${\mathrm{Ce}}^{3+}$ polarons [6,64,80]. Additionally, the double-integrated EPR intensity follows the expected defect chemistry. The ${\mathrm{CeO}}_{2-\delta }$ nanoparticles calcined at 400 °C exhibit the strongest envelope at g≈1.96, consistent with a higher density of near-surface oxygen vacancies that stabilize ${\mathrm{Ce}}^{3+}$ [6,64,80]. As the calcination temperature increases, the normalized intensity decreases overall for ${\mathrm{CeO}}_{2-\delta }$ nanoparticles calcined at 600–1000 °C, with a pronounced minimum at 600 °C (no resonance is resolved at room temperature under the same spectrometer settings used for the other samples). This behavior is consistent with the progressive healing and redistribution of oxygen-deficient sites in ${\mathrm{CeO}}_{2}$ [64,80]. Thus, two processes plausibly dominate during high-temperature calcination in air of the ${\mathrm{CeO}}_{2-\delta }$ nanoparticles: replenishment of lattice oxygen, which removes vacancy-trapped electrons, and grain growth, which lowers the surface-to-volume ratio and thereby the density of surface vacancy sites [64,80]. It is also worth noting that surface superoxide $O_{2}^{-}$ typically exhibits a rhombic *g* tensor with $g_{1}\approx 2.04$–2.05, $g_{2}\approx 2.01$–2.02, and $g_{3}\approx 2.00$, which produces an anisotropic powder pattern at room temperature [81]. Within our calcination series, a shift from isolated to clustered vacancy configurations naturally explains the faster decay of the EPR signal relative to bulk oxidation state metrics. In addition, signals from itinerant carriers would display Dysonian asymmetry, which is not observed. The absence of both the rhombicity characteristic of $O_{2}^{-}$ and the Dysonian line shapes characteristic of metallic conduction argues against these alternative assignments. Accordingly, EPR intensities need not correlate with bulk ${\mathrm{Ce}}^{3+}$/${\mathrm{Ce}}^{4+}$ ratios from XPS, because EPR detects only unpaired spins. Isolated oxygen vacancies localize two electrons on second-shell Ce cations and yield EPR-active ${\mathrm{Ce}}^{3+}$ polarons, whereas vacancy clusters redistribute charge and often pair electrons into states that are EPR-silent [82,83]. We therefore attribute the nearly symmetric resonance at g≈1.96, strongest at a calcination temperature of 400 °C, not resolved at 600 °C within our signal-to-noise ratio, and reappearing as a weaker signal at 800–1000 °C, to ${\mathrm{Ce}}^{3+}$ and vacancy-bound ${\mathrm{Ce}}^{3+}$ complexes. For instance, Chakarova et al. [84] reported that nanoparticles exhibiting {100} or {110} terminations frequently show a pronounced (g≈1.96) envelope from ${\mathrm{Ce}}^{3+}$–vacancy motifs, whereas {111}-dominated surfaces often display much weaker or absent signals at comparable ${\mathrm{Ce}}^{3+}$/${\mathrm{Ce}}^{4+}$ ratios. Conversely, on reduced ${\mathrm{CeO}}_{2}$(111), linear vacancy clusters are prevalent [83] and facilitate oxygen migration by vacancy hopping [85]. Furthermore, modest anisotropy has been reported for faceted ${\mathrm{CeO}}_{2}$, where both {100}- and {110}-terminated nanoparticles often display two features at ($g_{⊥}\approx 1.952$) and ($g_{\|}\approx 1.933$) that arise from ${\mathrm{Ce}}^{3+}$ [86,87,88]. In such cases, the relative intensities can track the facet-dependent vacancy concentration, with {100} generally exhibiting a stronger ${\mathrm{Ce}}^{3+}$ signal than {110}, consistent with our Raman and XPS indicators of higher vacancy density on {100}. By contrast, {111} surfaces tend to favor vacancy clustering and can be EPR-silent near (g≈1.96) even when XPS indicates comparable average ${\mathrm{Ce}}^{3+}$ content [88].

**Figure 6:**
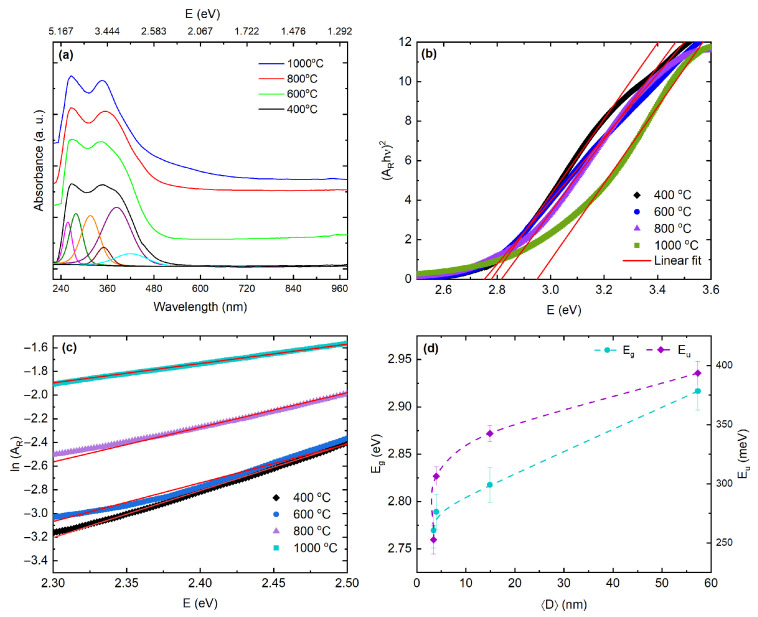
UV–Vis analysis of ${\mathrm{CeO}}_{2-\delta }$ nanoparticles calcined at 400, 600, 800, and 1000 °C. (**a**) Room-temperature diffuse-reflectance spectra plotted as reflectance–absorbance $A_{R}=-\log_{10}R$, 240–960 nm (≈1.29–5.17 eV). The deep-UV O 2p→Ce 4f charge-transfer band near ∼5.8 eV lies outside this window. (**b**) Direct-allowed Tauc plots, ${\left[A_{R}h\nu \right]}^{2}$ vs. E=hν; linear fits (5–60% of the ordinate within 2.0–6.2 eV) yield $E_{g}$ values used in Table 4. (**c**) Urbach analysis, $\ln A_{R}$ vs. *E*; slopes give ${\left(E_{u}\right)}^{-1}$. (**d**) Structure–property correlation: $E_{g}$ (left axis) and $E_{u}$ (right axis) versus crystallite size 〈D〉 from Rietveld line-profile analysis. $E_{g}$ increases with 〈D〉 while $E_{u}$ rises nearly monotonically. Dashed lines guide the eye; error bars are 1σ.

Therefore, the calcination-temperature-dependent attenuation of the g≈1.96 signal in the ${\mathrm{CeO}}_{2-\delta }$ nanoparticles, together with a minimum in intensity at 600 °C, suggests that surface structure further modulates the spin-active population. This behavior is consistent with partial oxygen replenishment and grain growth that reduce the population of isolated vacancy-bound ${\mathrm{Ce}}^{3+}$ polarons, along with a shift toward vacancy clustering that renders electrons EPR silent or strongly broadened, even when XPS still reports a finite average ${\mathrm{Ce}}^{3+}$ fraction [80,82,83,84,85,88]. These trends show that oxygen vacancies and ${\mathrm{Ce}}^{3+}$ polarons not only distort the lattice and modify local spin states but also reshape the electronic density of states near the band edges. Having characterized these defects structurally and electronically, we now assess their influence on the optical response. UV–Vis absorption is an appropriate tool for this purpose, since it responds both to the intrinsic band gap and to defect-related transitions introduced by nonstoichiometry. Figure 6a shows room-temperature UV–Vis spectra for ${\mathrm{CeO}}_{2}$ nanoparticles calcined between 400 and 1000 °C. The spectra were collected over 240–960 nm (≈1.29–5.17 eV), so any deeper charge-transfer feature near ∼5.8 eV lies outside this window and is inferred from the literature rather than directly resolved here [89,90,91]. Within the studied spectral range, deconvolution reveals two contributions near ∼3.8 and ∼3.4 eV. The ∼3.8 eV component corresponds to O 2p → Ce 4f charge transfer that shapes the fundamental edge, while the weaker ∼3.4 eV feature reflects ${\mathrm{Ce}}^{3+}$ 4$f^{1}$→ 5$d^{1}$ and vacancy-related states characteristic of oxygen-deficient ceria [89,90]. In colloidal dispersions, two UV bands near ∼245 nm (≈5.1 eV) and ∼330–350 nm (≈3.5–3.8 eV) are commonly reported, consistent with this assignment and with the presence of a deeper charge-transfer band at higher energy [91]. With increasing calcination temperature, the band that defines the absorption edge sharpens and slightly intensifies, consistent with crystallite growth and reduced microstrain that lower inhomogeneous broadening; these trends correlate with XRD peak narrowing and Raman $F_{2g}$ sharpening [89,90]. This evolution should not be interpreted as a confinement effect. In ${\mathrm{CeO}}_{2-\delta }$, the apparent onset is strongly influenced by vacancy- and ${\mathrm{Ce}}^{3+}$-related states and by tailing. Calcination in air coarsens the grains and partially reoxidizes the lattice, which reduces the spectral weight of 4f- and vacancy-related states near 3.3–3.6 eV and narrows the inhomogeneous edge; the resulting cleaner, more bulk-like onset yields a modest increase in $E_{g}$ even as the crystallites grow. A sub-gap tail extending from the visible into the near-IR range becomes more pronounced for the ${\mathrm{CeO}}_{2-\delta }$ nanoparticles calcined at 800 and 1000 °C. Although residual Mie scattering from larger aggregates can raise the long-wavelength baseline, the persistence of this tail, together with the ∼3.4 eV component, indicates a genuine electronic contribution from oxygen vacancies that convert ${\mathrm{Ce}}^{4+}$ to ${\mathrm{Ce}}^{3+}$ and produce F and $F^{+}$ centers [92], often accompanied by an increased Urbach energy [50].

To quantify the optical gap, we evaluated $E_{g}$ using three complementary estimators. First, Tauc analysis for a direct-allowed transition (linearity of ${\left(\alpha h\nu \right)}^{2}$ vs. hν near the onset) yields $E_{g}=2.778\pm 0.003$ eV for ${\mathrm{CeO}}_{2-\delta }$ nanoparticles calcined at 400 °C and 2.949±0.007 eV for those calcined at 1000 °C, with a shallow minimum at 600 °C followed by a net blue shift at higher temperatures [93,94,95]. Herein, it is worth noting that for ${\mathrm{CeO}}_{2}$ the direct versus indirect nature of the absorption onset remains debated. As shown in Figure 6b, indirect-allowed Tauc fits exhibit comparable linearity and yield gap values within $\Delta E_{g}\approx 0.1$–0.2 eV of the direct-allowed estimates at each temperature, indicating that the extracted $E_{g}$ is more sensitive to the assumed exponent than diagnostic of the transition type (here $\Delta E_{g}\equiv \left|E_{g}^{\mathrm{dir}}-E_{g}^{\mathrm{ind}}\right|$). In addition, the energy-equation method reproduces the same evolution within uncertainty, yielding 2.789±0.018, 2.770±0.018, 2.818±0.019, and 2.917±0.020 eV for ${\mathrm{CeO}}_{2-\delta }$ nanoparticles calcined at 400, 600, 800, and 1000 °C, respectively. By contrast, a derivative-based estimator that locates the steepest onset slope gives slightly larger absolute values and accentuates the high-temperature increase, reaching 3.30±0.05 eV for nanoparticles calcined at 1000 °C. The consistency in the temperature dependence across all methods indicates that the observed blue shift is robust and not an artifact of a particular fitting protocol, and the corresponding numerical values are summarized in Table 4. These results suggest that the blue shift mainly comes from edge sharpening as the grains grow and inhomogeneous broadening is reduced. Since EPR and XPS do not show an increase in the ${\mathrm{Ce}}^{3+}$ donor population, any Moss–Burstein contribution is likely small. Additionally, the Raman spectra exhibit no D or G bands, and the XPS data reveal only adventitious carbon together with a minor carbonate component typical of air-exposed ${\mathrm{CeO}}_{2}$. These observations indicate that the ∼3.4 eV feature and its associated tail arise from vacancy–${\mathrm{Ce}}^{3+}$ states rather than from residual carbon. Furthermore, sub-gap disorder and tail states were quantified by the Urbach energy $E_{u}$ using the absorbance-like ordinate $A_{R}$. In the sub-edge region we assume an exponential form $A_{R}\left(E\right)=A_{0}\exp\left(E/E_{u}\right)$, which implies $\ln A_{R}=\ln A_{0}+E/E_{u}$ and $E_{u}={\left[d(\ln A_{R})/dE\right]}^{-1}$ in the linear regime [50]. The extracted values are 352.7 meV, 306.4 meV, 342.5 meV, and 393.5 meV for ${\mathrm{CeO}}_{2-\delta }$ nanoparticles calcined at 400, 600, 800, and 1000 °C, respectively (Table 4, Figure 6c,d). This nonmonotonic trend reflects two partially decoupled effects. First, reduced microstrain as crystallites coarsen sharpens the fundamental edge, which lowers $E_{u}$ between calcination temperatures of 400 and 600 °C. Second, vacancy-related localized states and enhanced electron–phonon coupling in the coarsened oxide increase the density of tail states at higher temperatures, which raises $E_{u}$ again at 800–1000 °C even as $E_{g}$ widens. The shallow minimum at 600 °C therefore marks the cleanest band edge in this series. We also estimated the refractive index from the Moss relation, $n^{4}E_{g}=C$ with C=95eV, giving $n={\left(C/E_{g}\right)}^{1/4}$ [96]. Because this relation is empirical and less reliable for oxides, we treat *n* as a trend indicator rather than an absolute index. Using the direct-allowed Tauc gaps in Table 4 and propagating their uncertainties yields n=2.456±0.005, 2.462±0.005, 2.448±0.005, and 2.420±0.006 for ${\mathrm{CeO}}_{2-\delta }$ nanoparticles calcined at 400, 600, 800, and 1000 °C, respectively. These values are consistent with spectroscopic ellipsometry reports for ${\mathrm{CeO}}_{2}$, which place the refractive index between n≃2.3 and 2.4 over 550–633 nm [97,98,99]. Those studies show that *n* increases with film density and texture [98] and tends to cluster around 2.33±0.08 at 632.8 nm [99]. Compared with these literature values, our Moss-derived indices decrease slightly as $E_{g}$ widens across the calcination series, which is consistent with weak normal dispersion and with a reduction in electronic polarizability as defects anneal. Because Moss estimates are heuristic and wavelength-independent, the absolute *n* we obtain also folds in dispersion and effective-medium effects in nanoparticle powders, such as porosity, packing, and surface states. The fact that our values agree with ellipsometric data to within a few percent supports the picture that calcination-driven densification and band-edge sharpening lower the polarizability in a way that is consistent with previous optical-constant studies.

Here, the UV–Vis metrics plotted against crystallite size 〈D〉 show a consistent structure–property trend. As 〈D〉 increases with calcination, the band edge blue-shifts slightly and the Moss-estimated refractive index decreases, consistent with reduced electronic polarizability as the gap widens. The Moss values, which range from n=2.456±0.005 to 2.420±0.006 for ${\mathrm{CeO}}_{2-\delta }$ nanoparticles calcined at 400 and 1000 °C, respectively, fall within literature ranges for ${\mathrm{CeO}}_{2}$ near 550–633 nm and are used here only as trend indicators, since the absolute *n* also depends on dispersion and effective-medium effects in nanoparticle powders. In contrast, the Urbach energy $E_{u}$ shows a shallow minimum at intermediate 〈D〉 and then increases again for the largest crystallites, while the ∼3.4 eV shoulder and the visible sub-gap tail remain present. This separation between the behavior of $E_{g}$ and $E_{u}$ points to two defect families that evolve differently: microstructural disorder, which anneals as the crystallites coarsen and sharpens the edge, and vacancy-derived states, which stay active or become stabilized at high temperature. The collapse of these trends onto the 〈D〉 axis in Figure 6d indicates that crystallite size is the key control parameter linking band-edge alignment and the density of tail states.

To further quantify these correlations, we regressed $E_{u}$ and $E_{g}$ against the ${\mathrm{Ce}}^{3+}$ fraction and the microstrain 〈ε〉. Even with n=4 samples, linear fits still reveal clear tendencies: $E_{u}$ correlates strongly with ${\mathrm{Ce}}^{3+}$$\left(1.89\pm 0.62\;\mathrm{meV}\;\mathrm{per}\;\%;\;r=0.91\right)$ and shows no statistically resolved dependence on 〈ε〉$\left(-13.6\pm 77.3\;\mathrm{meV}\;\mathrm{per}\;\%;\;r=-0.12\right)$, consistent with tailing dominated by vacancy–4f complexes. In contrast, $E_{g}$ increases monotonically with 〈D〉$\left(\left(3.40\pm 0.36\right)\times 10^{-3}\;\mathrm{eV}\;{\mathrm{nm}}^{-1};\;r=0.99\right)$, shows only a moderate correlation with ${\mathrm{Ce}}^{3+}$$\left(0.0040\pm 0.0022\;\mathrm{eV}\;\mathrm{per}\;\%;\;r=0.79\right)$, and a weak to moderate anticorrelation with 〈ε〉 that is not statistically significant at the 1σ level $\left(-0.142\pm 0.162\;\mathrm{eV}\;\mathrm{per}\;\%;\;r=-0.53\right)$. Taken together, these trends are consistent with a scenario in which vacancy populations control the near-edge tail density, whereas the fundamental onset responds mainly to reduced disorder and to the loss of near-edge defect weight during annealing. Two considerations help reconcile the positive $E_{g}$ trend with the ${\mathrm{Ce}}^{3+}$ fractions in Table 2. First, XPS is surface-sensitive on the scale of a few nanometers, whereas diffuse-reflectance UV–Vis probes the optical bulk, so high-temperature sintering can enrich near-surface ${\mathrm{Ce}}^{3+}$ while the bulk becomes cleaner. Second, the microstrain parameter reflects long-wavelength fields and is only weakly sensitive to localized vacancy clusters, which are the defects that most effectively generate band-tail states. With only n=4 data points, higher-order multivariate models are ill-conditioned, so the reported univariate slopes, standard errors, and Pearson *r* values represent the appropriate level of inference. A more complete experimental determination of n(ω) or of the complex dielectric function would require ellipsometry on dense films or absolute reflectance with Kramers–Kronig analysis. While such measurements lie outside the present scope, the trends identified here already provide a quantitative description for connecting vacancy chemistry, microstructure, and near-edge optical properties in ${\mathrm{CeO}}_{2-\delta }$ nanoparticles.

## 4. Conclusions

We have clarified how grain growth and vacancy chemistry govern the near-edge optical response of nanocrystalline ${\mathrm{CeO}}_{2-\delta }$ obtained from a chymosin-assisted Pechini route. Calcination between 400 and 1000 °C yields phase-pure fluorite ${\mathrm{CeO}}_{2}$ with crystallite sizes from ∼3.4 to ∼57 nm and microstrain decreasing from 0.79% to 0.05%. The size–strain scaling and grain-growth kinetics follow a normal growth law with exponent m=2 and activation energy $Q\approx 155\;\mathrm{kJ}\;{\mathrm{mol}}^{-1}$, indicating curvature-driven, interface-controlled grain-boundary migration in this nanocrystalline regime. Raman spectroscopy evidences strain relief and a reduction of defect-rich terminations, while XPS and EPR together show that surface ${\mathrm{Ce}}^{3+}$ and vacancy-bound polarons evolve nonmonotonically with temperature, reflecting oxygen uptake and defect clustering. Diffuse-reflectance UV–Vis measurements reveal a modest blue shift of the apparent band gap from $E_{g}\approx 2.78$ to 2.95 eV as crystallites coarsen, whereas the Urbach energy $E_{u}$ grows with increasing ${\mathrm{Ce}}^{3+}$ content and sub-gap tailing. Correlation analysis thus separates the roles of size, strain, and ${\mathrm{Ce}}^{3+}$: vacancy populations control the density of tail states, while the fundamental onset responds mainly to strain relaxation and reduced near-edge defect weight. These insights provide a microscopic basis for tuning defect-mediated optical and transport properties in ${\mathrm{CeO}}_{2-\delta }$ nanoparticles and related oxide systems.

## Figures and Tables

**Table 1:** Unit cell lattice parameters (a=b=c), volume (*V*), chi-square ($\chi^{2}$), average crystallite size (〈D〉), and microstrain (〈ε〉), estimated by Rietveld refinement of the XRD pattern. Microstrain is reported as 〈ε〉(%).

Parameter	Calcination Temperature (∘C)			
	400	600	800	1000
a=b=c (Å)	5.41489 (4)	5.41398 (3)	5.41188 (2)	5.41337 (2)
*V* (${Å}^{3}$)	158.77 (4)	158.69 (3)	158.51 (2)	158.64 (2)
〈D〉 (nm)	3.43 (4)	3.98 (2)	14.9 (3)	57.3 (6)
〈ε〉(%)	0.79 (2)	0.17 (3)	0.39 (8)	0.05 (1)
$\chi^{2}$	1.06	1.08	1.15	1.13

**Table 2:** Binding energies and relative ${\mathrm{Ce}}^{3+}$/${\mathrm{Ce}}^{4+}$ contents for ${\mathrm{CeO}}_{2-\delta }$ nanoparticles calcined at 400–1000 °C.

Calcination Temp. (°C)	3$d_{5/2}$ (eV)						3$d_{3/2}$ (eV)					${\mathrm{Ce}}^{3+}$ (%)	${\mathrm{Ce}}^{4+}$ (%)	${\mathrm{Ce}}^{3+}$/${\mathrm{Ce}}^{4+}$
	$\nu_{0}$	ν	$\nu^{\prime }$	$\nu^{\prime \prime }$	$\nu^{\prime \prime \prime }$		$u_{0}$	u	$u^{\prime }$	$u^{\prime \prime }$	$u^{\prime \prime \prime }$			
400	882.42	883.30	887.24	889.33	898.33		898.53	900.99	901.89	907.96	916.77	35.44	64.56	0.55
600	882.25	883.04	885.68	889.05	898.28		898.30	900.78	901.21	908.15	916.87	9.84	90.16	0.11
800	882.27	883.73	888.28	890.06	897.63		898.40	900.79	902.28	907.73	916.61	41.61	58.39	0.71
1000	883.16	882.19	888.10	889.81	897.46		898.43	900.75	902.37	907.74	916.61	49.54	50.45	0.98

**Table 3:** O 1s peak positions, relative oxygen fractions, and stoichiometric ratios *x*, $x^{\prime }$, and $\Delta =x-x^{\prime }$ for ${\mathrm{CeO}}_{2-\delta }$ nanoparticles calcined at 400–1000 °C.

Calcination Temp. (°C)	$O_{\mathrm{Latt}}$ (eV)	$O_{\mathrm{OH}}$ (eV)	$O_{H_{2}O}$ (eV)	$O_{\mathrm{Latt}}$ (%)	*x*	$x^{\prime }$	Δ
400	528.57	529.10	530.90	41.73	1.82	2.27	−0.45
600	529.43	529.86	531.09	27.27	1.87	2.09	−0.22
800	529.39	530.11	531.17	51.14	1.79	2.40	−0.61
1000	529.32	529.97	531.46	43.00	1.75	2.20	−0.45

**Table 4:** Optical parameters of ${\mathrm{CeO}}_{2-\delta }$ nanoparticles as functions of calcination temperature: band gap ($E_{g}$), refractive index (*n*), and Urbach energy ($E_{u}$).

Calcination Temp. (°C)	Optical Band Gap $E_{g}$ (eV)			*n*	$E_{u}$ (meV)
	Tauc’s Plot	Energy Equation	Derivative		
400	2.778±0.003	2.7892±0.0184	2.88±0.03	2.4563±0.0054	352.7
600	2.752±0.001	2.7695±0.0183	2.87±0.04	2.4621±0.0054	306.4
800	2.816±0.003	2.8176±0.0186	3.03±0.04	2.4480±0.0054	342.5
1000	2.949±0.007	2.9166±0.0201	3.30±0.05	2.4199±0.0056	393.5

## Data Availability

The data supporting this study are available upon appropriate request, in accordance with the institutional policies of CAPES, CNPq, and UFS.
